# Supplementary material for: Controlled nonlinear magnetic damping in spin-Hall nano-devices
Source: Nat Commun. 2019 Nov 18;10:5211. doi: 10.1038/s41467-019-13246-7 (PMC6861234; doi:10.1038/s41467-019-13246-7)
Supplement: Supplementary file 1 — Supplementary Information [file 41467_2019_13246_MOESM1_ESM.pdf]

## Supplementary information

# Controlled nonlinear magnetic damping in spin-Hall nano-devices

Boris Divinskiy[1], Sergei Urazhdin[2], Sergej O. Demokritov[1], and Vladislav E. Demidov[1*]

[1]*Institute for Applied Physics and Center for Nonlinear Science, University of Muenster, 48149 Muenster, Germany*

[2]*Department of Physics, Emory University, Atlanta, GA 30322, USA*

[*]Corresponding author. E-mail: demidov@uni-muenster.de

**Supplementary Note 1. Determination of the critical currents.**

According to the spin-transfer torque theory, injection of spin current into ferromagnets results not only in the compensation of the natural damping, but also in enhancement of magnetic fluctuations[1]. At a certain critical current value $I_C$, natural damping becomes completely compensated, while the intensity of fluctuations diverges. At currents $I$ below $I_C$ the inverse of the fluctuation intensity exhibits a linear dependence on current, extrapolating to zero at $I=I_C$. This dependence provides a precise method for the determination of $I_C$ by the BLS spectroscopy, whose high sensitivity allows one to detect magnetic fluctuations even at $I=0$. Supplementary Figure 1 shows the inverse of the measured integral BLS intensities of current-dependent fluctuations for the Py and CoNi disks. As expected, the data for both types of samples exhibit a linear dependence on current, yielding $I_C=16$ mA for Py, and 14.5 mA for CoNi.

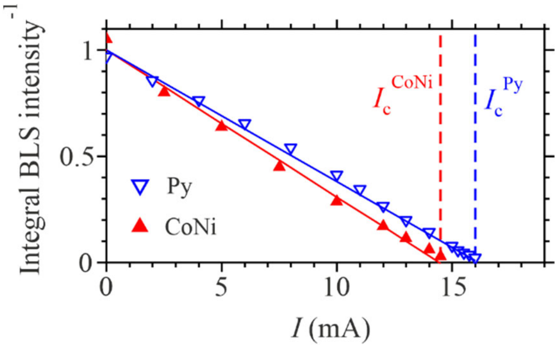


**Supplementary Figure 1. Enhancement of magnetic fluctuations by spin current.** Normalized current dependences of the inverse of the integral BLS intensity for the Py and CoNi disks, as labelled. Symbols: experimental data, line: linear fit. $I_C$ marks the extrapolated value of current, at which the intensity of fluctuations is expected to diverge. The data were obtained at $\mathbf{H_0}$=2 kOe.

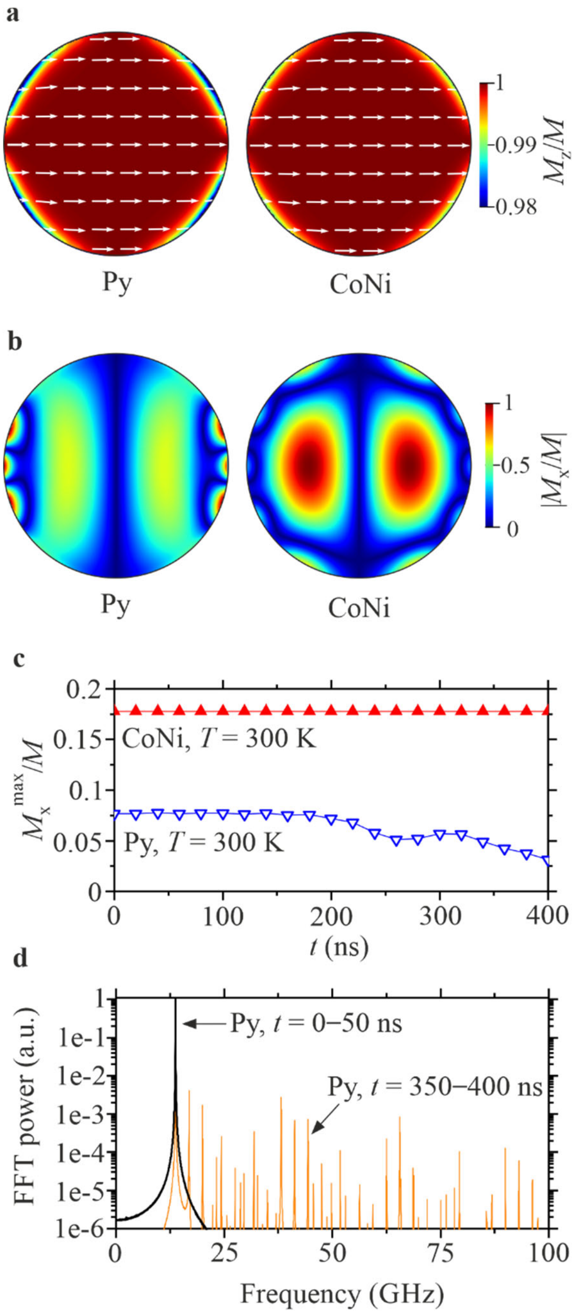


**Supplementary Figure 2. Results of micromagnetic simulations for magnetic disks. a**, Spatial distributions of the static magnetization. **b**, Spatial distributions of the dynamic magnetization for the dynamic mode with the lowest frequency. **c**, Temporal evolution of the free precession amplitude starting with a large initial amplitude at *t*=0. The simulations were performed with negligible linear damping, emulating the damping compensation by the spin current. *T*=300 K. **d**, Fourier spectra of magnetization oscillations in the Py disk before (*t*=0-50 ns) and after (*t*=350-400 ns) the onset of nonlinear damping. Simulations were performed for disks with the diameter of 0.5 μm.

**Supplementary Note 2. Effects of residual precession ellipticity**

As seen from the data of Supplementary Figure 3a, auto-oscillations become noticeably suppressed by the nonlinear damping in two samples, where the PMA anisotropy differs from the saturation magnetization by about 10% in one or the other direction. In both samples, the oscillation intensities significantly decrease, and the maximum intensity is achieved at larger currents. These behaviors are consistent with our interpretation. In particular, the current value, at which the maximum intensity is achieved, is determined by two factors: (i) the value of the intensity of magnetization oscillations necessary for the onset of strong nonlinear relaxation and (ii) the rate, at which the intensity increases with the increase of current above the threshold value. The intensity (i) depends on the efficiency of the nonlinear coupling determined by the degree of precession ellipticity. In agreement with this picture, the samples with larger ellipticity exhibit an onset of strong nonlinear relaxation at smaller intensities. The rate (ii) is also expected to depend on the efficiency of nonlinear mechanisms. Since nonlinear scattering counteracts the energy flow due to the injection of the pure spin current, in samples with larger ellipticity, the rate (ii) should be smaller, in agreement with the data of Supplementary Figure 3a. Finally, in samples with very large ellipticity, such as the Py sample (Fig. 2 in the main text), the strong nonlinear relaxation develops at very small intensities at currents close to the threshold current, resulting in complete suppression of auto-oscillations.

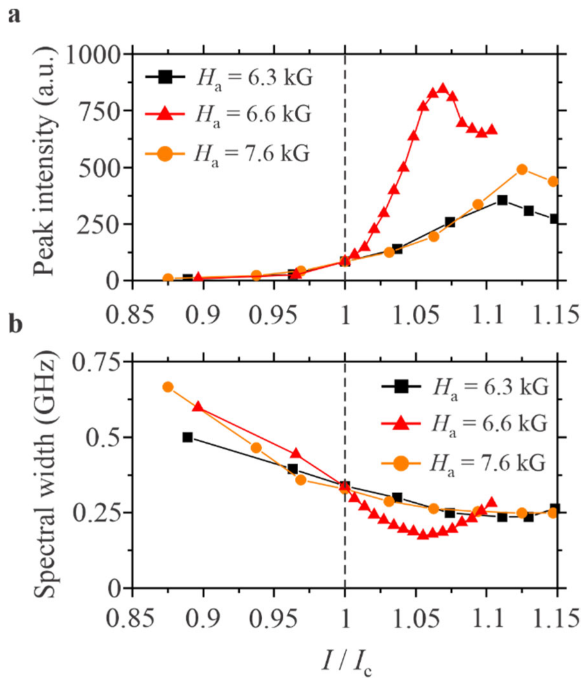


**Supplementary Figure 3. Effects of residual precession ellipticity.** Transition to the auto-oscillations in CoNi disks with different PMA anisotropy fields, as labelled. **a**, Maximum intensities of the BLS spectra vs current. **b**, Current dependences of the spectral width of the BLS peaks at half the maximum intensity. Symbols: experimental data, lines: guides for the eye. The data were obtained at $\mathbf{H_0}$=2 kOe

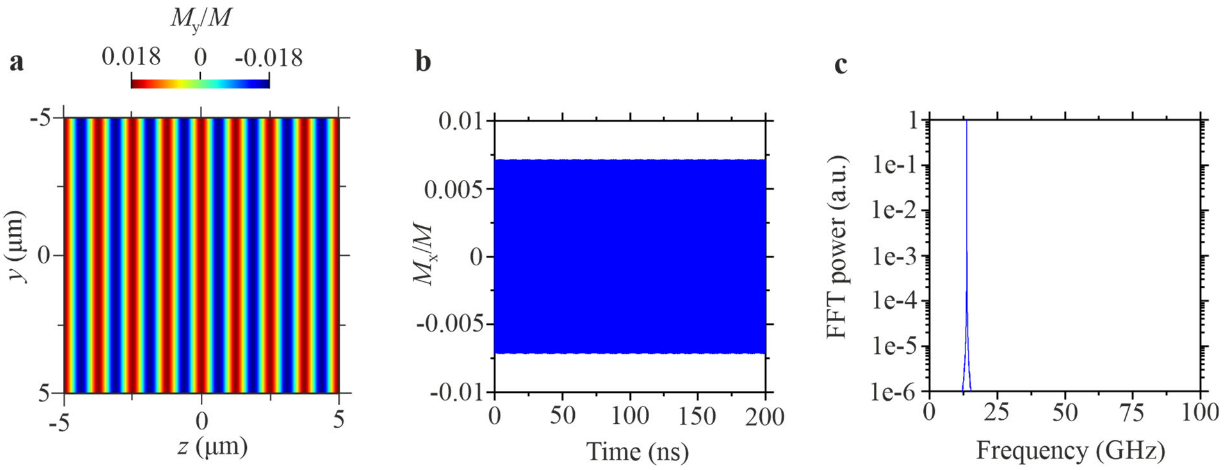


**Supplementary Figure 4. Procedure used for the calculations of the spin-wave spectra. a**, Magnetic moments are initially deflected from their equilibrium orientation in the *y*-direction by a small angle. The deflection is sinusoidal with the period corresponding to the selected wavevector **k**. **b**, The free dynamics of magnetization is calculated for artificially small Gilbert damping parameter. **c**, By performing the Fourier transform of the obtained temporal trace, the frequency corresponding to the wavevector **k** is determined. By varying the spatial period of the initial deflection pattern and the deflection angle, the dependence of the frequency of spin waves on the wavevector and the amplitude is determined.

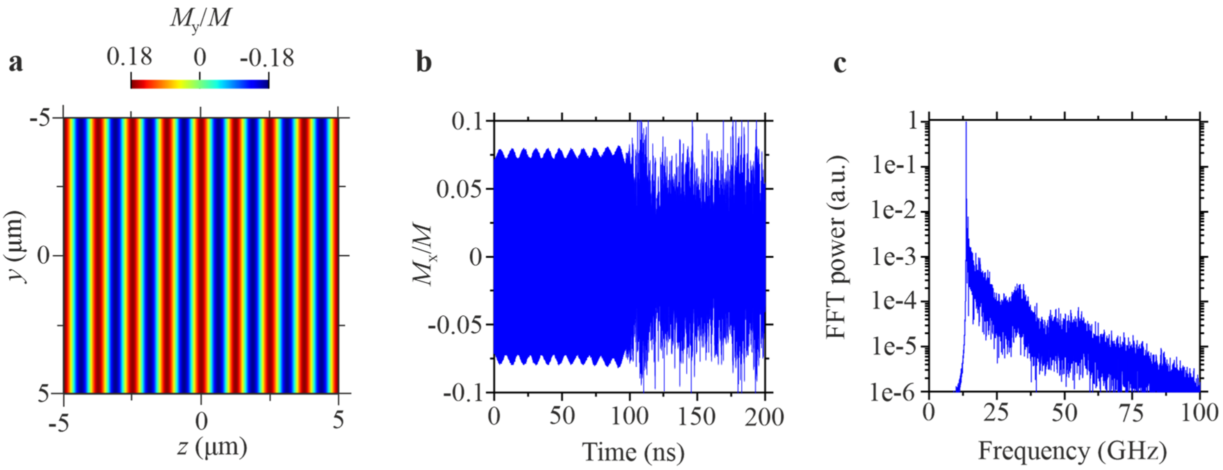


**Supplementary Figure 5. Analysis of the instability at large precession angles. a**, Initial state with the selected spatial period and a relatively large deflection angle is defined. **b**, The free dynamics exhibits a transition from periodic oscillations to the multimodal regime. **c**, Fourier transform calculated using the entire calculated time interval exhibits a broad spectrum (compare to the Supplementary Figure 4c). By performing Fourier transform of 50 ns-long intervals at different delays (see Fig. 5d in the main text), the time-dependent flow of energy from the initially excited mode to other modes due to the nonlinear coupling is analyzed. The temporal evolution of the amplitudes of specific modes (see Fig. 5c in the main text) is determined by analyzing the corresponding Fourier harmonics.

## Supplementary References

1. Slavin, A. & Tiberkevich, V. Nonlinear Auto-Oscillator Theory of Microwave Generation by Spin-Polarized Current. *IEEE Trans. Magn.* **45**, 1875-1918 (2009).
